# Predicting Intraindividual Change in Satisfaction with Life During COVID-19: A Prospective Study of Swiss Older Adults with Differing Levels of Childhood Adversity

**DOI:** 10.1007/s10902-024-00791-2

**Published:** 2024-07-25

**Authors:** Myriam V. Thoma, Florence Bernays, Joffrey Fuhrer, Jan Höltge, Aileen N. Salas Castillo, Shauna L. Rohner

**Affiliations:** 1 Psychopathology and Clinical Intervention, Institute of Psychology, University of Zurich, Binzmühlestrasse 14/17, CH-8050 Zurich, Switzerland; 2 University Research Priority Program “Dynamics of Healthy Aging”, University of Zurich, Zurich, Switzerland; 3 Chair of Human Resource Management and Leadership, Institute of Business Administration, University of Zurich, Plattenstrasse 14, 8032 Zurich, Switzerland; 4 Swiss Center for Affective Sciences, University of Geneva, Geneva, Switzerland; 5 University of Hawaii at Mānoa, Honolulu, HI USA; 6 Competence Centre for Mental Health, School of Health Sciences, OST – Eastern Switzerland University of Applied Sciences, 9001, St. Gallen, Switzerland

**Keywords:** Intraindividual change, Satisfaction with life, Growth curve modeling, Meaning in life, Adverse childhood experiences

## Abstract

*Objectives* This study examined intraindividual change in satisfaction with life (SWL) in Swiss older adults before, during, and after COVID-19. It assessed whether predictors of adaptation influenced SWL stability, and whether this differed depending on adverse childhood experiences (ACE). *Methods* SWL was assessed eight times over a 21-month period. ACE, emotion regulation, meaning in life, and subjective socio-economic status (SES) were assessed as predictors. Data were analyzed using growth curve modeling. *Results* The sample consisted of two groups: A risk group (RG: *n* = 111, *M*_*age*_ = 69.4 years) comprised of individuals with a high risk of having been exposed to ACE, and a (low-risk) control group (CG: *n* = 120, *M*_*age*_ = 70.3 years). Intraindividual change in SWL was predicted by (presence of) meaning in life only in the RG, and by subjective SES only in the CG. *Conclusion* Results identified predictors of stable SWL trajectories and the potential for positive psychological functioning into later life, despite past and current prolonged adversity.

**Supplementary Information:**

The online version contains supplementary material available at 10.1007/s10902-024-00791-2.

## Introduction

The coronavirus disease 2019 (COVID-19) pandemic began to impact Switzerland in late February 2020 and escalated quickly afterwards (Giachino et al., 2020). Within days, in mid-March, the Federal Council of Switzerland declared an “extraordinary situation” for Switzerland. Switzerland's heightened state of alert was particularly evident in the subsequent mobilization of thousands of military reserves to assist in the COVID-19-related crisis management, a measure that was unprecedented since the era of World War II (Giachino et al., 2020). As advanced age and related factors (i.e., more chronic health conditions) were linked to a greater risk for hospitalization, severe health consequences, and higher mortality rates during this period; older adults were considered to belong to a particularly vulnerable age group in need of higher protection. For many older adults, COVID-19 and the related protective measures led to prolonged periods of social isolation, distress, and fear; in addition to stressful economic and psychosocial consequences (see Parlapani et al., 2021).

Given such prolonged negative emotional and affective states, older adult’s mental health symptoms and positive psychological functioning could be expected to meaningfully worsen over the course of the COVID-19 pandemic. Empirical support for this was provided globally, as well as in Switzerland (Macdonald & Hülür, 2021), with studies on older adults reporting decreased quality of life, positive affect, and sleep quality; and increased levels of anxiety, depression, and negative affect (De Pue et al., 2021; Hansen et al., 2022; Zaninotto et al., 2022). However, a considerable number of studies did not find evidence of a substantial change in older adults’ health (Parlapani et al., 2021; Prati & Mancini, 2021). Some research reported stability (i.e., non-deterioration) on various indices of positive psychological functioning, such as self-rated (mental) health, satisfaction with life (SWL), and well-being (e.g., Kivi et al., 2021; Schäfer et al., 2020; Wettstein et al., 2022). As such, although older adults were expected to belong to a particularly vulnerable group during the COVID-19 pandemic, a non-negligible line of research highlights the potential for (relatively) stable positive psychological functioning.

Classic (although not aging-specific) theories of stability, such as the ‘hedonic treadmill’ (Brickman & Campbell, 1971), or the ‘set-point theory’ (Lykken & Tellegen, 1996), may be considered to explain such findings. The common denominator of these theories (for an overview see Luhmann & Intelisano, 2018) is the assumption that human beings have a general propensity to (continuously) adapt to an unstable (i.e., potentially stressful) environment and maintain a relatively stable ‘set point’ in well-being (allowing for short-term intraindividual fluctuations). However, research has shown a wide heterogeneity in older adult’s health and well-being in response to stressful life events (e.g., Bonanno & Mancini, 2012; Prati & Mancini, 2021). Therefore, to better understand such interindividual differences and promote positive psychological functioning in later life, it is necessary to identify predictors of (in-)stability.

In later life, these predictors may be shaped by the varied life experiences and coping strategies acquired over the lifetime. For example, exposure to adverse childhood experiences (ACE) has been identified as a crucial factor for long-term health outcomes, with negative consequences believed to accumulate over the life span (Dannefer, 2003). For instance, ACE has been linked to a poorer adaptation to stress, increased sensitivity to future stress, and a higher volatility in psychological functioning in later life (e.g., McEwen, 2012). ACE has also been shown to impact factors linked to individual coping, such as emotion (dys-)regulation, which may influence the ability to maintain the (relative) stability of well-being in stressful contexts. For example, a clinical trial for posttraumatic stress disorder (PTSD) found that emotion regulation difficulties mediated the relationship between ACE and PTSD symptoms, depression, and poor physical health (Cloitre et al., 2019).

With regard to positive psychological functioning, a related explanatory factor is ‘meaning in life’, defined as judging, feeling, and evaluating one’s life as imbued with purpose, coherence, and significance (e.g., Martela & Steger, 2016). Meaning in life has been associated with numerous positive outcomes, such as decreased risk of mortality and cardiovascular events, improved general health, and reduced suicidal thoughts (e.g., Cohen et al., 2016). Having meaning in life is also believed to be crucial in dealing with traumatic experiences and adversities. For example, a meta-analysis by Fischer and colleagues (2020) revealed that having meaning in life was a predictor of adaptive responses to traumatic events among US military personnel with posttraumatic stress symptoms. However, there has been little investigation into whether meaning in life affects the stability of well-being over time in the context of prolonged adversity, such as the COVID-19 pandemic.

Socio-economic aspects also represent key factors for well-being in times of stress or adversity (e.g., Kivi et al., 2021). For instance, research by Thoma et al. (2021a) in older adults with welfare-related ACE and age-matched controls showed that those with ACE had lower education and income levels, and lower satisfaction with their financial situation and socio-economic status (SES). These socio-economic factors also explained the significant health and stress disparities between the two groups (Thoma et al., 2021a). Recent studies on older adult’s ability to adapt to COVID-19 also reinforce the importance of socio-economic factors for well-being. For instance, research from the English Longitudinal Study of Ageing identified socio-economic inequalities linked to health and well-being during the COVID-19 pandemic. While the higher socio-economic groups showed overall better mental health, the decrease in quality of life was smaller for those in the poorest wealth group (Zaninotto et al., 2022). However, as this is a relatively new and emerging research area, further studies are required to identify predictors of (in-)stability in this high-risk, yet heterogenous, population of older adults during the COVID-19 pandemic.

The current study is embedded within a larger longitudinal project on differential ageing trajectories in high-risk individuals with past experiences of early-life adversity. The first face-to-face assessment (i.e., baseline) for the project started in July 2019 and lasted until December 2019. This was followed by five planned contact interviews over the phone, separated by three months each, over the course of 18 months. After this, a second face-to-face assessment (i.e., follow-up) was originally planned. However, due to the tightening of COVID-19 measures in December 2020 and January 2021, there was a temporary prohibition of conducting face-to-face assessments with high-risk (e.g., older) individuals at the study site (i.e., University of Zürich, Switzerland). As a result, the study protocol had to be amended as the follow-up would have taken place from January 2021. The amended study protocol, which was approved by the responsible ethics committee, included a delayed start of three months for the follow-up assessment, as well as the inclusion of a sixth contact interview between the fifth (and originally last) contact interview and the (delayed) start of the follow-up. As the vast majority of studies on the psychological impact of the COVID-19 pandemic draws on cross-sectional evidence (see Parlapani et al., 2021), the time-line of this study provided a unique opportunity to examine the longer-term adaptation to COVID-19, including pre-, peri-, and post-data. This information is particularly limited with regard to older individuals. Given the far-reaching and long-term social, psychological, economic, and cultural consequences of the COVID-19 pandemic (e.g., Settersten et al., 2020); it was not only opportune, but also necessary to further research on this topic, with a view to the potential benefits for society that could be derived from the data.

### Institutional Setting

Switzerland has a robust social security system comprising five branches: Old-age, survivors’, and invalidity insurance; health and accident insurance; compensation for loss of earnings due to military or alternative civilian service, or maternity; unemployment insurance; and family allowances (The Federal Council, 2024). According to the Federal Office of Public Health (2024), health insurance in Switzerland is compulsory, universal, and provided by non-profit insurers. The basic, affordable coverage includes illness, maternity, and accidents. All benefits and services are provided equally to all insured individuals, with no discrimination based on health status or other indicators. Switzerland has long been recognized for its excellence in healthcare. As noted in the Euro Health Consumer Index, “Switzerland has enjoyed a solid reputation for excellence in healthcare for a long time” (Björnberg & Phang, 2019, p. 9). The particularities of the institutional setting in Switzerland are assumed to play a meaningful role in shaping the experiences and well-being of Swiss older adults. The universal and impartial nature of the healthcare system, combined with inclusive social security measures, may contribute to a higher baseline of well-being and resilience among the elderly population in Switzerland.

### Study Aims and Hypotheses

This prospective study aimed to examine intraindividual change in positive psychological functioning in a sample of Swiss older adults, before, during, and after COVID-19. Specifically, this study examined whether intraindividual change in positive psychological functioning (indexed by SWL) could be predicted based on between-person differences in relevant predictors. Chosen on the basis of current evidence, the following predictors were assessed: The number of ACE, emotion regulation, meaning in life, and subjective SES. It was hypothesized (hypothesis 1) that a more beneficial manifestation of the predictors (i.e., lower number of ACE, more adaptive emotion regulation, higher meaning in life, higher subjective SES) would be linked to more stable trajectories in SWL over time (i.e., fewer within-person changes from an individual’s set point). Furthermore, it was hypothesized that the influence of the predictors on the trajectories of SWL over time would differ depending on the level of exposure (i.e., high vs. low) to ACE (hypothesis 2). To test this hypothesis, two groups of older individuals with differing risks of exposure to ACE were compared with each other.

## Material and Methods

### Study Design

This study was conducted with the written informed consent of all participants. The protocols of the study (ID: 19.4.3; ID: 20.12.24) were approved by the Ethics Committee of the Faculty of Arts and Social Sciences in the University of Zürich, Switzerland.

### Participants and Recruitment

Participants were native Swiss-German speakers, aged 50 or older. The sample was comprised of two groups: a risk group (RG), composed of individuals affected by compulsory social measures and placements (CSMP) in Switzerland (for a minimum of one year) up until age 18, and a control group (CG) of non-affected participants. The term ‘risk’ refers to the high risk of exposure to early-life adversity as a minor in the context of the experienced CSMP, as documented in a diverse set of biographical, historic, and scientific publications (e.g., Kuhlmann et al., 2013; Leuenberger & Seglias, 2008; Thoma et al., 2021b).

Recruitment took place between July and December 2019. Most RG participants were contacted using a list of affected individuals who were interested in participating in research studies, which was provided by the Swiss Federal Office of Justice. Additional recruitment efforts included contacting individuals who spoke publicly about their CSMP experiences and word-of-mouth between participants. The CG was also recruited using flyers and advertisements on websites, in public places, older adult-specific spaces, as well as through researchers and contacts of the study team.

The final sample consisted of *N* = 231 participants (RG: *n* = 111; CG: *n* = 120). The overall proportion of missing data was 5.8%, with no more than 10% missing for any of the items. Little’s test indicated that missings were completely at random (*X*^2^ = 30.08, *p* = 0.931), which justified the use of growth curve modelling (Curran et al., 2010).

### Data Collection

**Fig. 1:**
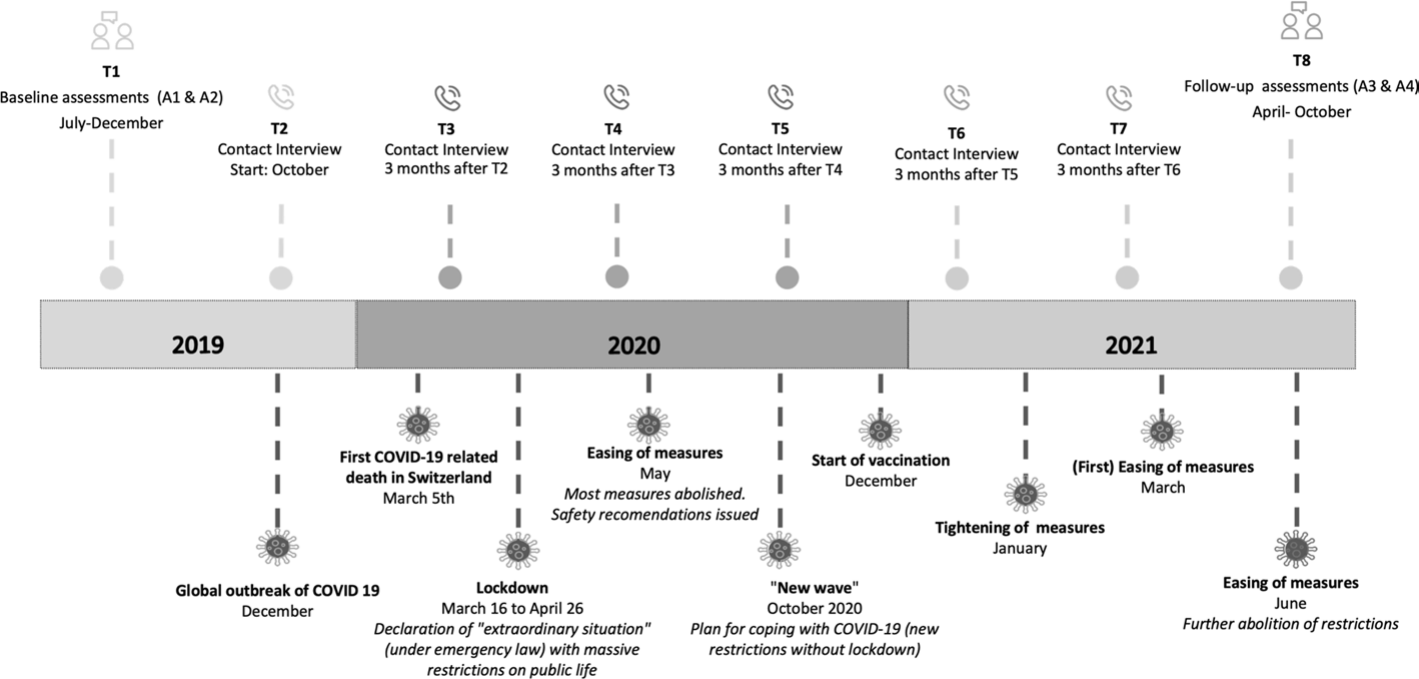
Timeline of study alongside the COVID-19 development and measures in Switzerland

Interested individuals completed a screening process to establish eligibility and receive study information. Eligible participants took part in four main face-to-face assessments (baseline, T1: A1 and A2; follow-up, T8: A3 and A4), each with a maximum duration of two hours. Figure 1 shows an illustration of the study data collection time points alongside the timeline of COVID-19 measures in Switzerland. The face-to-face assessments (A1-A4) were either conducted at the psychological institute of the university or at the participant’s home. Between these baseline and follow-up assessments, an additional six contact interviews (T2-T7) were conducted via telephone at three-month intervals. These telephone interviews had a maximum duration of 30 min and collected data on current physical and mental health, stress, and SWL.

At baseline (T1: July to December 2019), mental health disorders were assessed at A1 using a structured clinical interview. Information on the CSMP experiences was also collected for the RG. At the end of A1, participants received a set of questionnaires to complete at home and return at A2, which was conducted within seven days. At A2, data was collected on health information, functional and cognitive abilities, well-being, psychological resources, and stress experiences. After the baseline assessment, participants received a monetary compensation (approximately $250) and a list of psychological support options.

At follow-up (T8: April to October 2021), socio-demographic information, potentially stressful life events (experienced since baseline), and current and past-year mental health disorders were assessed at A3. At the end of A3, participants received a set of questionnaires to be completed at home and returned at A4. At A4, data was collected on cognitive status, functional abilities, stress experiences, and psychological states and traits. After the follow-up assessment, participants again received a monetary compensation (approximately $250) and a list of psychological support options.

### Measures

The measures that were assessed in and used for the current study are presented in the following sections. Table 1 presents an overview of the assessment time points, including all content that was assessed in the study (see Table 1).

**Table 1:** Overview of assessment time points across the study and content of data collected

Assessment	Information collected
T1. Baseline Face-to-face (A1)	Socio-demographic information, mental health disorders, and the experiences with the compulsory social measures and/or placements for the risk group. Informed consent
T1. Baseline Face-to-face (A2)	Health information, functional and cognitive abilities, well-being, psychological resources, and stress experiences (including childhood trauma)
T2-T7 Contact interviews (by telephone)	Current physical and mental health, stress, and satisfaction with life
T8. Follow-up Face-to-face (A3)	Socio-demographic information, potentially stressful life events since baseline, and current and past-year mental health disorders
T8. Follow-up Face-to-face (A4)	Cognitive status, functional abilities, stress experiences (including adverse childhood experiences), and psychological states and traits

*T* Time point (T1-T8), *A* Assessment (A1-A4)

#### Socio-Demographics

Socio-demographics were assessed via self-report questionnaires, with additional questions for the RG regarding their CSMP experiences (e.g., reason, duration). Age and gender were used as control variables in the prediction of within-SWL change.

#### Adverse Childhood Experiences

Early life adversities were assessed using the German version of the *Adverse Childhood Experiences Questionnaire* (ACE-Q, Felitti et al., 1998; Wingenfeld et al., 2011). The ACE-Q is a brief rating scale (10 dichotomous items) measuring the participant’s recollections of exposure to different types of abuse, neglect, and household dysfunction. The higher the score, the higher the number of adversities experienced (range: 1 – 10). The ACE-Q shows acceptable reliability (*α* = 0.74 in the current study).

#### Emotion Regulation

The German version of the *Emotion Regulation Questionnaire* (ERQ; Gross & John, 2003) was used to assess emotion regulation strategies. The ERQ measures the preferred strategy to regulate emotions, either by cognitive reappraisal (*k* = 6) or by expressive suppression (*k* = 4). Ten items are rated on a seven-point Likert scale, ranging from 1 (strongly disagree) to 7 (strongly agree). Scores range from 6 to 42 for cognitive reappraisal and from 4 to 28 for expressive suppression. In the current study, the subscales showed acceptable to high internal consistency (reappraisal: $\alpha$ = 0.81; suppression: $\alpha$ = 0.75).

#### Meaning in Life

The German version of the *Meaning in Life Questionnaire* (MLQ; Steger et al., 2006) assessed participant’s psychological state of meaning in life. The MLQ consists of two subscales: “Presence of Meaning” (five items) and “Search for Meaning” (five items). The 10 items are rated on a seven-point Likert scale ranging from 1 (absolutely untrue) to 7 (absolutely true). Subscale scores range from 5 to 35, with higher scores indicating that participants feel that their life has a valued meaning and purpose (Presence), and that participants are actively searching for something or someone that will give their life meaning or purpose (Search). Both subscales showed high internal consistency in the current study (presence: *α* = 0.87; search: *α* = 0.95).

#### Subjective Socio-Economic Status

The German version (adapted for use in Switzerland) of the single-item *MacArthur Scale of Subjective Social Status* (Adler et al., 2000; Hoebel et al., 2015) was used to assess subjective SES. This scale measures a person’s perceived rank relative to others on a symbolic “ladder”, based on common SES indicators. The score corresponds to the number of the rung on the ladder (1–10) on which the participant placed their “X” (range: 1–10). The higher the selected rung, the higher the perceived social standing.

#### Satisfaction with Life

The German version of the *Satisfaction with Life Scale* was used an index for positive psychological functioning (Diener et al., 1985; Glaesmer et al., 2011). It consists of five items rated on a seven-point Likert scale. Scores range from 5 to 35, with higher scores indicating higher SWL. In the current study, this scale showed high internal consistency across all measurement occasions (*α*_T1_ = 0.89, *α*_T2_ = 0.86, *α*_T3_ = 0.87_,_ *α*_T4_ = 0.85, *α*_T5_ = 0.86, *α*_T6_ = 0.86, *α*_T7_ = 0.87, *α*_T8_ = 0.90).

## Data Analysis

Growth curve modeling (R Studio version 4.2.1, package “nlme”) was used to examine the influence of the predictors on the within-person change in SWL over time. Following the recommendations of modeling within-person change (Curran et al., 2010), participants who completed fewer than three out of eight SWL surveys were excluded from the analysis (RG: *n* = 10; CG: *n* = 2). See Table 1 in the supplementary material for a comparison of excluded and included participants. The recommendations for the use of growth curve modeling were met (Boscardin et al., 2022). The growth curve models were fitted separately for the RG and CG given that a growth curve based on the pooled sample would assume that the model parameters are equal across the two samples (Bollen & Curran, 2006; Curran et al., 2010). Following earlier studies (e.g., Mroczek & Spiro, 2005), each between-person factor (i.e., Level-2 variables) was tested separately as a predictor of change in SWL (i.e., Level-1 variable). To explore whether Level-2 predictors influence within-person change in SWL, interaction terms were computed for ‘time’ (scored as 1–8 for T1–T8) and Level 2 predictors (i.e., ACE, emotion regulation, meaning in life, subjective SES). Level 2 predictors and numeric covariates (i.e., age) were grand-mean centered separately for each group, such that each score reflects the extent to which an individual deviates from the overall group mean of the RG or CG. This approach is recommended when examining how between-person factors influence within-individual trajectories (Enders & Tofighi, 2007). As such, the intercept reflects the expected outcome for a participant at the overall sample mean of a given group, with deviations from the intercept illustrating how strongly individuals deviate from the overall sample mean.

At Level 1, the model was conceptualized as follows:

$${SWLS DEV}_{ij}= {\pi }_{0i}+ {\pi }_{1j}\left({time}_{ij}\right)+ {{\pi }_{1j}\left({time}_{ij}\right)}^{2}+ {\varepsilon }_{ij.}$$

The main outcome (i.e., SWL DEV) represents how strongly the levels of SWL of participant *i* deviate from their individual average SWL at time *j*. Consequently, positive values in SWL DEV indicate that individuals experienced more SWL at time* j* than they experienced on average across T1 to T8. Hence, we compared an individual’s level of SWL at time *j* with their average levels of SWL across all eight measurement points. This approach provides a more representative operationalization of average SWL than when using only a single measurement as an indicator of average SWL. The score thus reflects positive or negative deviations from participant’s individual set point of SWL at time *j.* To ensure that the results were robust across age and gender, both variables were controlled for when predicting within-change.

Missing data was handled using restricted maximum likelihood (REML). The alpha level was set at 0.05 unless otherwise specified, while a p-value below 0.10 was considered to indicate a statistical trend.

## Results

### Sample Description

A total of 260 participants were recruited (RG: *n* = 135; CG: *n* = 125), with* N* = 243 completing the baseline assessment (RG: *n* = 121; CG: *n* = 122). Table 2 illustrates the sample size, response rates, and average SWL per measurement for each group. The final sample, after the exclusion of incomplete cases, consisted of *n* = 111 in the RG and *n* = 120 in the CG. The sample had an average age of 70.4 years (RG:* M* = 71.4, *SD* = 11.7; CG: *M* = 71.9, *SD* = 9.5), 53.9% identified as male (RG: 59%; CG: 49.6%), and the majority (42.9%) were married (RG: 38.1%; CG: 48.7%).

**Table 2:** Sample size and mean satisfaction with life levels at each time measurement, separately for the risk and control groups

Measurement	Risk group				Control group				Group comparisons
	*M*	*SD*	*n*	*RR*	*M*	*SD*	*n*	*RR*	
Time 1	4.17	1.49	121	100%	5.01	1.38	122	100%	*t*(242) = 4.48**
Time 2	4.36	1.45	121	100%	5.28	1.18	119	97%	*t*(239) = 5.46 **
Time 3	4.45	1.46	115	95%	5.39	1.05	116	95%	*t*(230) = 5.70**
Time 4	4.65	1.39	119	98%	5.30	1.14	120	98%	*t*(238) = 3.92**
Time 5	4.60	1.36	114	94%	5.32	1.09	118	97%	*t*(231) = 4.41**
Time 6	4.72	1.36	109	90%	5.30	1.07	115	94%	*t*(223) = 3.50**
Time 7	4.59	1.42	111	92%	5.28	1.14	119	97%	*t*(229) = 4.00**
Time 8	4.28	1.45	105	87%	5.02	1.42	115	94%	*t*(219) = 3.83**

*M* mean, *SD* standard deviation, *RR* Response rate per measurement occasion based on T1 sample size. *Time 1* Baseline assessment, with all subsequent timepoints representing 3-month intervals

*p < 0.05, **p < 0.001

Table 3 provides an overview of the study variables by group. Overall, it was found that the RG reported significantly more ACE (*d* = 0.47), higher expressive suppression (*d* = 0.26), and were less satisfied with their SES (*d* = 0.38).

**Table 3:** Overview of study variables and group comparisons

*Predictors*	Risk group (*n* = 111)		Control group (*n* = 120)		Group comparisons
	*M*	*SD*	*M*	*SD*	
Adverse childhood experiences	6.0	4.5	2.3	2.1	*t*(171) = − 8.31, *p* < 0.001**
Emotion regulation—cognitive reappraisal	28.3	7.0	27.3	7.5	*t*(234) = − 1.03, *p* = 0.333
Emotion regulation—expressive suppression	17.4	5.7	14.5	5.4	*t*(232) = − 4.08, *p* < 0.001**
Meaning in life—presence	23.4	5.4	23.2	4.5	*t*(203) = − 0.31, *p* = 0.749
Meaning in life—search	19.8	9.9	21.9	9.1	*t*(219) = 1.59, *p* = 0.110
Subjective socio-economic status	5.0	2.0	6.50	1.6	*t*(207) = 6.03, *p* < 0.001**
Age	71.4	11.7	71.9	9.5	*t*(230) = 0.04, *p* = 0.964
Gender (% male)	59.0%			49.6%	*X*^*2*^ = 50.24*, p* = 0.239

Test statistics represent results from two-sided t-tests

*M* mean, *SD* standard deviation

^*^p < 0.05; ** *p* < 0.001

### Intra- and Interindividual Change in Satisfaction with Life Trajectories

Model selection procedures showed that SWL was nested within individuals and that accounting for the hierarchical structure (participants nested in time) was necessary (RG: ICC (1) = 0.68, *F*(123, 794) = 16.99, *p* < 0.001; ICC (2) = 0.94; CG = ICC (1) = 0.71, *F*(123, 821) = 20.70, *p* < 0.001; ICC (2) = 0.95). The final model for the RG was a random slope model (*X*^2^(*2*) = 37.90, *p* < 0.001, AIC_intercept + slope_ = 2017, AIC_intercept_ = 2051, BIC_intercept + slope_ = 2051, BIC_intercept_ = 2070), with a quadratic effect in intraindividual change in SWL (*B* = -4.52, SE = 0.73, *p* < 0.001), including a first-order autoregressive structure (*X*^2^(1) = 7.01, *p* = 0.008). The final model for the CG was a random slope model (*X*^2^(*2*) = 45.90, *p* < 0.001, AIC_intercept_ + slope = 1630, AIC_intercept_ = 1672, BIC_intercept + slope_ = 1664, BIC_intercept_ = 1696), with a quadratic effect in intraindividual change in SWL (*B* = -3.20, SE = 0.58, *p* < 0.001). The model for the CG had no autoregressive structure as including autoregressive effects improved the model fit only in the RG but not in the CG (*X*^2^ (1) = 2.95, *p* = 0.085).

**Fig. 2:**
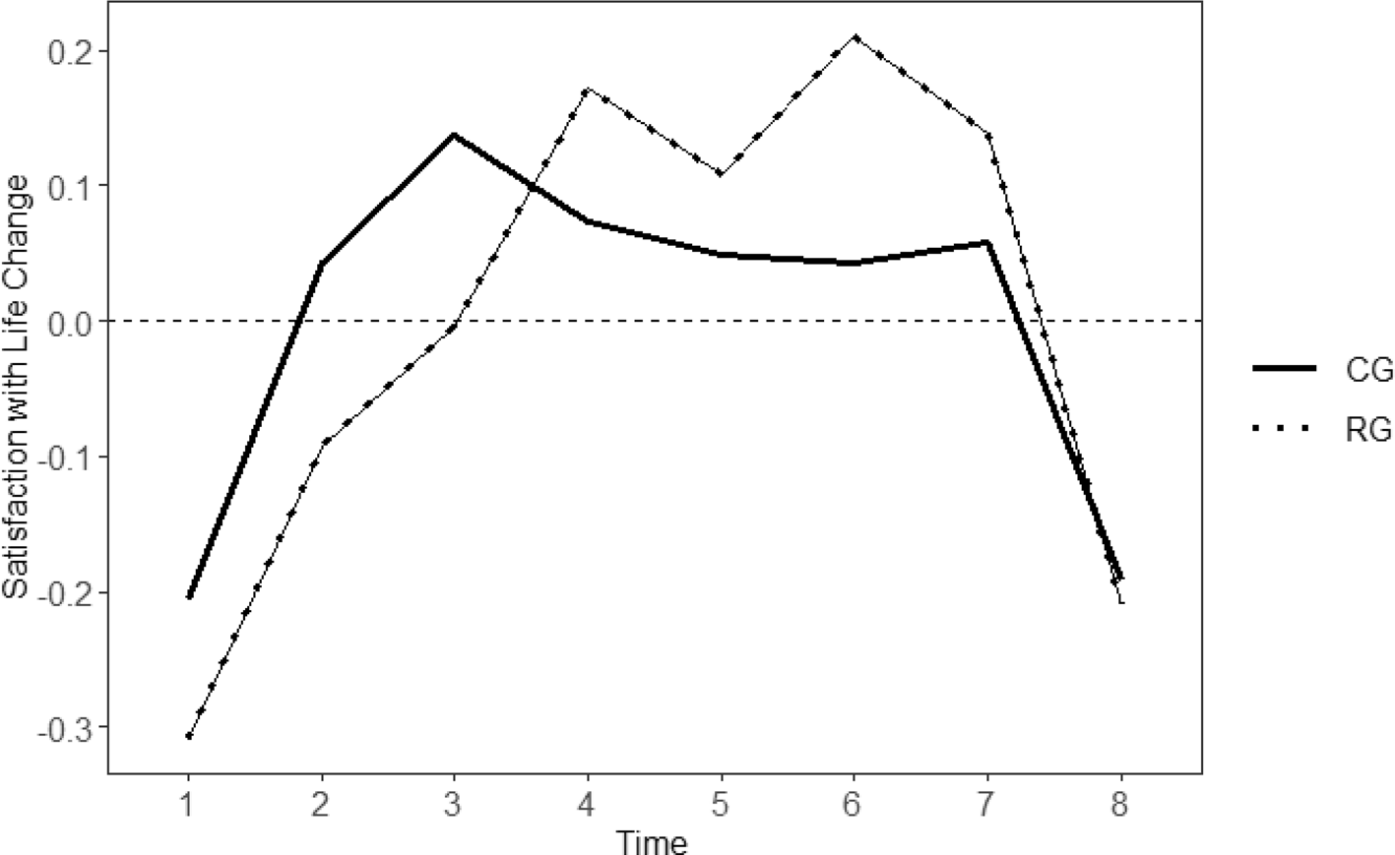
Trajectories of intraindividual change in satisfaction with life for the risk and control groups. Note: The horizontal dashed line (y = 0) reflects no deviation from the average level of satisfaction with life (SWL); positive values indicate positive deviations from the average level of SWL, negative values indicate negative deviations from the average level of SWL. CG = Control Group; RG = Risk Group; Time = timepoints represent 3-month intervals

These models indicated that SWL changed significantly across time in both groups (RG: *B* = -4.59, *SE* = 0.64, *p* < 0.001; CG: *B* = -3.24, *SE* = 0.53, *p* < 0.001), suggesting that participants varied in the amount of curvature of their within-person SWL trajectories. Figure 2 illustrates the extent to which SWL changed in both groups across the eight measurement points. When comparing change in SWL between the two groups at each time point, change in SWL was shown to differ significantly between the two groups at T6 (*t*(222) = 2.20, *p* = 0.028). The RG (*M* = 0.21, *SD* = 0.62) showed stronger positive deviations from their individual set point of SWL than the CG (*M* = 0.04, *SD* = 0.45). As such, equivalence of model parameters between the two groups could not be assumed, suggesting that it was necessary to fit growth curve models separately for the two samples.

### Predictors of within-Person Change

As SWL was shown to change significantly within individuals across time and across groups, it was then tested whether between-person differences in ACE, emotion regulation, meaning in life, and subjective SES would predict intraindividual change in SWL.

#### Adverse Childhood Experiences

When testing between-person differences in the number of ACE as a predictor, results indicated that the number of ACE did not significantly predict within-person change in SWL (RG: *B* = 0.10, *SE* = 0.21, *p* = 0.608; CG: *B* = -0.30, *SE* = 0.19, *p* = 0.108). Results are shown in Table 2 in the supplementary material.

#### Emotion Regulation

Regarding ‘cognitive reappraisal’, no significant effect was found in the RG (*B* = 0.15, *SE* = 0.09, *p* = 0.105), nor in the CG (*B* = 0.12, *SE* = 0.07, *p* = 0.098). Regarding ‘expressive suppression’, no significant effects were found (RG: *B* = −0.16, *SE* = 0.12, *p* = 0.197; CG: *B* = 0.03, *SE* = 0.11, *p* = 0.705). Post-hoc unpaired t-tests revealed no significant differences in change in SWL between participants with high and low cognitive reappraisal in the CG (*p* > 0.05). Results are shown in Table 3 in the supplementary material.

#### Meaning in Life

**Fig. 3:**
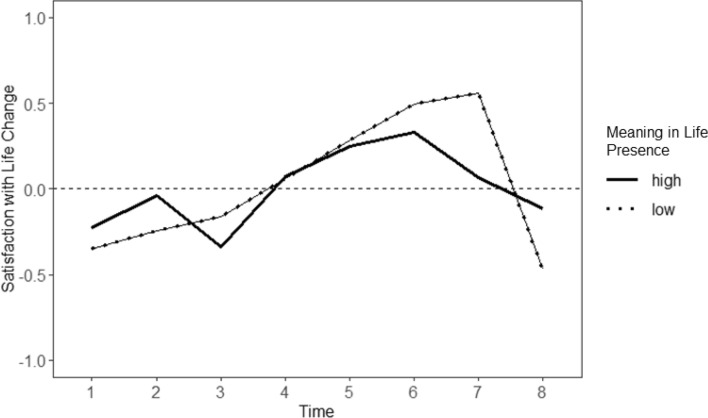
Trajectories of intraindividual change in satisfaction with life for the risk group based on (the presence of) meaning in life. Note: The horizontal dashed line (y = 0) reflects no deviation from the average level of satisfaction with life (SWL); positive values indicate positive deviations from the average level of SWL; negative values indicate negative deviations from the average level of SWL. High and low levels refer to one SD above and below the mean of Meaning in Life–Presence; Time = timepoints representing 3-month intervals

When testing meaning in life as a predictor of within-person SWL trajectories (see Table 4), a trend was observed in which meaning in life ‘presence’ predicted intraindividual change in SWL across time in the RG (*B* = 0.22, *SE* = 0.12, *p* = 0.066), with no significant effect found in the CG (*B* = 0.18, *SE* = 0.12, *p* = 0.134). The intraindividual change in SWL in the CG was plotted at one standard deviation above and below the mean of meaning in life ‘presence’ (Fig. 3). Participants in the RG who reported a high ‘presence’ of meaning in life showed more stable levels of SWL across time. This pattern was found for six measurements (except T3 and T4). This effect was particularly pronounced at T8, as those with a low ‘presence’ of meaning in life showed a sharp decrease from their individual SWL set point, while those with a high ‘presence’ of meaning in life showed only a minimal decrease. Post-hoc unpaired t-tests revealed that participants in the RG with higher meaning in life ‘presence’ showed (marginally) significantly less change in SWL at T7 (*t*(99) = 1.62, *p* = 0.053, *M* = 0.12, *SD* = 0.73) than those with lower levels of meaning in life ‘presence’ (*M* = 0.34, *SD* = 0.61).

**Table 4:** Intraindividual change in satisfaction with life predicted by meaning in life

Fixed effects	Risk group (*n* = 111)			Control group (*n* = 120)		
	Estimates	*SE*	95% CI	Estimates	*SE*	95% CI
Intercept	− 0.01	0.03	− 0.05; 0.05	− 0.01	0.03	− 0.05; 0.04
Slope	− 4.26	0.84	− 5.92; − 2.59	− 3.13	0.75	− 4.62; − 1.64
Time × age	0.12*	0.05	0.01; 0.24	− 0.10	0.06	− 0.21; 0.01
Time × gender	0.12	1.33	− 2.50; 2.75	0.27	1.08	− 1.84; 2.39
Time × meaning in life—presence	0.24*	0.12	0.00; 0.48	0.18	0.12	− 0.05; 0.43
Time × meaning in life—search	0.12	0.06	− 0.01; 0.25	− 0.08	0.06	− 0.19; 0.03
Random effects						
Intercept		0.57*	0.44; 0.74		0.44	0.34; 0.56
Slope		0.12*	0.10; 0.16		0.09	0.07; 0.12
Residual		0.68			0.53	
− 2LL		− 918			− 765	
AIC		1877			1568	

Risk Group:* n* 824 observations (Level 1), Control Group: *n* 877 observations (Level 1), *SE* standard errors, *95% CI* 95% confidence intervals, *-2LL* 2 Log likelihood, *AIC* Akaike’s Information Criterion

^*^p < 0.05

#### Subjective Socio-Economic Status

**Fig. 4:**
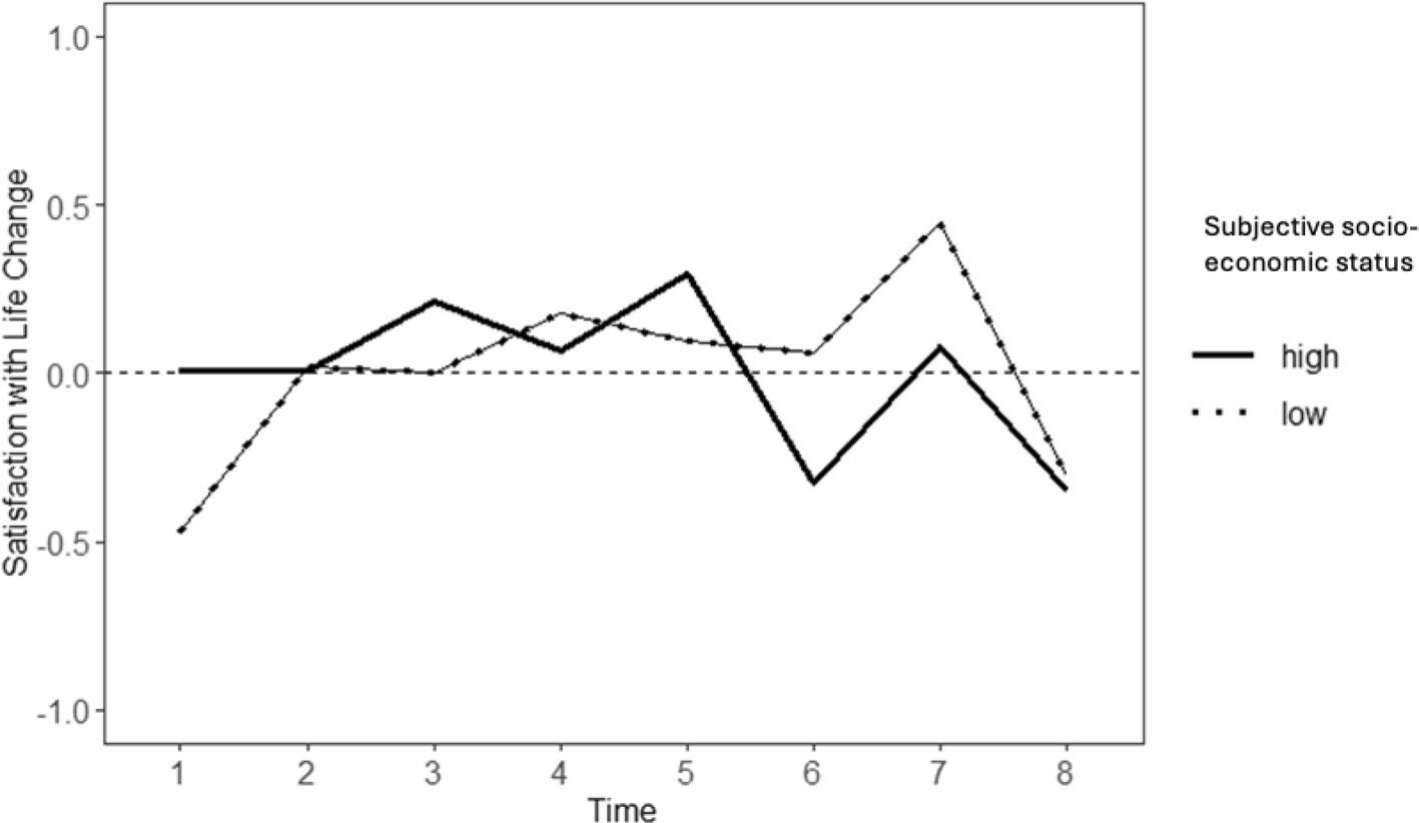
Trajectories of intraindividual change in satisfaction with life for the control group based on subjective socio-economic status. Note: The horizontal dashed line (y = 0) reflects no deviation from one’s average level of satisfaction with life (SWL); positive values indicate positive deviations from the average level of SWL; negative values indicate negative deviations from the average level of SWL. High and low levels refer to one SD above and below the mean of subjective socio-economic status; Time = timepoints representing 3-month intervals

Results showed that between-person differences in subjective SES predicted intraindividual change in SWL across time only in the CG (*B* = 0.73, *SE* = 0.30, *p* = 0.018), and not in the RG (*B* = 0.42, *SE* = 0.34, *p* = 0.218) (see Table 5). The intraindividual change in SWL in the CG was plotted at one standard deviation above and below the mean of subjective SES (see Fig. 4). At T1, those with low subjective SES showed lower levels of SWL than their individual set point, while those with high subjective SES showed no deviation from their average SWL levels. From T2 to T5, those with high (compared to low) subjective SES showed more positive deviations from their individual set point of SWL. This pattern reversed between T5 and T6, with those with high subjective SES showing a sharp decrease in SWL, while those with low subjective SES showed no such strong decline from their average SWL. Between T6 and T7, both groups (i.e., high and low subjective SES) showed an increase in SWL, while SWL decreased again from T7 and T8. Post-hoc unpaired t-tests revealed that participants in the CG with higher levels of subjective SES showed significantly less change in SWL at T7 (*t*(117) = 1.71, *p* = 0.044,* M* = 0.02, *SD* = 0.52), and (marginally) significantly less change in SWL at T4 (*t*(118) = 1.43, *p* = 0.077,* M* = 0.04, *SD* = 0.35), than those with lower levels of subjective SES (T7: *M* = 0.51, *SD* = 0.91; T4: *M* = 0.15, *SD* = 0.50).

**Table 5:** Intraindividual change in satisfaction with life predicted by subjective socio-economic status

Fixed effects	Risk group (*n* = 111)			Control group (*n* = 120)		
	Estimates	*SE*	95% CI	Estimates	*SE*	95% CI
Intercept	− 0.01	0.14	− 0.29; 0.27	0.01	0.13	− 0.25; 0.29
Slope	− 12.35*	4.45	− 21.09; − 3.61	3.57	4.20	− 4.67; 11.82
Time × age	0.11	0.06	− 0.01; 0.22	− 0.10	0.05	− 0.22; 0.01
Time × gender	0.67	1.31	− 1.91; 3.26	0.60	1.09	− 1.54; 2.75
Time × SES	0.49	0.32	− 0.13; 1.12	0.73*	0.30	0.12; 1.33
Random effects						
Intercept		0.46	0.36; 0.60		0.44*	0.34; 0.56
Slope		0.09	0.09; 0.12		0.09*	0.07; 0.12
Residual		0.68			0.53	
−2LL		− 985			− 818	
AIC		2004			1670	

Risk group:* n* 824 observations (Level 1), Control group: *n* 944 observations (Level 1), *SE* standard errors, *95% CI* 95% confidence intervals, *-2LL* 2 Log likelihood, *AIC* Akaike’s Information Criterion

^*^p < 0.05

## Discussion

This study examined intraindividual change in SWL in a sample of Swiss older adults, before, during, and after COVID-19. Specifically, it assessed the impact of various predictors on SWL and tested whether this impact differed depending on the level of exposure to ACE. Results showed that individuals meaningfully differed with respect to how strongly they deviated from their group mean SWL across time. While (the presence of) meaning in life predicted intraindividual change in SWL only in the risk group, subjective SES predicted intraindividual change in SWL only in the control group. These findings partly support the hypotheses of the current study. On one hand, (the presence of) meaning in life and higher subjective SES predicted intraindividual change in SWL (in support of hypothesis 1), which differed depending on the level of ACE exposure (i.e., high vs. low risk) (in support of hypothesis 2). On the other hand, the lower number of ACE and more adaptive emotion regulation were not found to have a meaningful impact on the intraindividual change in SWL in either group (failing to support hypotheses 1 and 2).

These findings add to the literature on the heterogeneity of older adults with regard to health, well-being, and adaptation to stressful life events (e.g., Galatzer-Levy & Bonanno, 2012), by providing evidence for interindividual differences in the intraindividual SWL change over the course of COVID-19. Both groups showed positive deviations from their average SWL during the middle phase of COVID-19, with negative deviations before and after. The reason why these deviations from the average SWL happened at the first and last measurement point is unclear and can only be speculated. It may be that the first and last SWL measurements were conducted in a face-to-face setting and the other measurements were completed by telephone, the negative deviations at the first and last assessment may reflect methodological differences. The deviations could also reflect potential feelings of stress or uncertainty within participants that may accompany the start and end of a study (and the researcher-participant relationship). While the risk group appeared to adapt more slowly at first to COVID-19, these individuals showed greater positive deviations from their average SWL compared to the control group. It may be that older adults with previous experiences of adversity may be better able to positively adapt and maintain well-being over time in the context of prolonged adversity.

The results on the predictors may shed further light on this finding, for instance, suggesting that individuals who have a greater sense of meaning in their lives may be more likely to maintain stability in their SWL during adverse times. This is consistent with results of a meta-analysis on meaning-centered therapies, which found that psychotherapeutic approaches focused on meaning in life were linked to improved quality of life and reduced psychological stress (Vos & Vitali, 2018). In the current study, having a sense of meaning in life had a stronger effect on SWL in the risk compared to the control group. It may be that for individuals who have not experienced traumata, the effect of meaning in life may be weaker as other variables that influence the stability of SWL have not come under threat and may exert a greater (positive) effect, such as self-compassion, self-esteem, or emotional stability. However, finding meaning in life might be more important for SWL in those with traumatic experiences. Traumata directly undermine one’s sense of meaning in life as it can disrupt one’s sense of purpose, coherence, and significance. This can prompt individuals to search for and restore a sense of meaning in their life, which may act as a strong protective factor for stable SWL. Research has also found that the search for meaning in life can be positively correlated with sadness, fear, and depression, and negatively correlated with SWL (e.g., He et al., 2023). However, given the non-significant findings on the search for meaning in life in the current study, future research should explore this further in older adult samples.

The finding that higher subjective SES predicted less intraindividual change in SWL, though only in the control group, is in line with existing COVID-19 research. For instance, in a Swedish longitudinal study, it was found that older individuals who expressed more COVID-19 related socio-economic worries had a lower level of well-being (Kivi et al., 2021). Similarly, in the longitudinal cohort study by Zaninotto et al. (2022), those in the higher socio-economic groups showed overall better mental health before and during COVID-19. Taken together with the results of the current study, these findings may suggest that having a higher subjective SES is indicative of a higher financial stability, which may function as a stress-buffer in times of economic insecurity, such as during COVID-19. Together, the results of this study indicate that policy and practice initiatives should focus on supporting more vulnerable individuals with lower (subjective) SES and aim to foster psychosocial supports to help older adults attain or maintain meaning in life. This may help to promote optimal levels of life satisfaction in older adults in Switzerland, now and in the case of future health crises (Hupkens et al., 2018; Toussaint et al., 2021).

Several strengths can be highlighted from the current study. The older mean age of the sample was a strength of this study, as older individuals remain a comparatively neglected age group in mental health research. Another strength was the use of two groups that differed regarding their exposure to ACE, particularly given the limited investigations with disadvantaged or at-risk individuals in high-income countries (Parlapani et al., 2021). Finally, a critical particularity of this project was that this prospective study began before the pandemic, with repeated measurements during and after. While an event such as the pandemic could not have been anticipated, the flexible adaptation of the study provided the unique opportunity to observe intraindividual change over the course of a naturalistic global stressor.

The study had several limitations. The first limitation is the implicit assumption that all participants were equally affected by COVID-19. Furthermore, future studies should also assess the impact of personality, given that certain personality traits (i.e., neuroticism) have previously been linked to higher volatility in SWL (Headey & Muffels, 2018). The retrospective assessment of ACE via self-report is an additional limitation of this study. Furthermore, the whole sample consisted of self-selected participants, which limits the generalization of the study’s findings. Furthermore, the risk group constitutes a very particular group of survivors of ACE who share a unique historical background. Most of the participating survivors were recruited via a list of contacts provided by the Federal Office of Justice (2024), which included research-interested individuals who had previously applied for the solidarity contribution. It may be that those survivors exhibit distinct characteristics, behaviors, and experiences, compared to survivors who did not apply for the solidarity contribution or who were not interested in partaking in a research project. In addition, it must be noted that most participants who completed less than three SWL surveys, and were thus excluded from the analyses (*n* = 10), belonged to the risk group. This might be due to the increased vulnerability of the survivors, as past studies have shown that individuals affected by compulsory social measures and placements suffer from more mental health and physical disorders than an age-matched control group (Thoma et al., 2021a, 2021b). Lastly, it is important to highlight that the Swiss social security system may provide a particularly supportive environment, which could have mitigated some of the negative impacts of the COVID-19 pandemic. The predictors examined in this study (e.g., meaning in life and subjective socio-economic status) may have different magnitudes of influence in countries with less supportive institutional settings. Future studies should consider examining these predictors in diverse socio-economic contexts to better understand the generalizability of the findings and the varying impacts of social support systems on psychological resilience during a meaningful period of (global) stress or adversity.

Together, the results show that despite the vulnerability status of older adults during the COVID-19 pandemic, older Swiss individuals demonstrated the potential to adapt and show positive psychological functioning, as indicated by positive deviations from their average levels of SWL. Key predictors of more stable SWL trajectories included higher (presence of) meaning in life, particularly for those with high exposure to ACE, as well as higher subjective SES. Such findings challenge the traditional deficits-based approach to health and aging and emphasizes the potential for positive psychological functioning into later life, even in the context of prolonged adversity.

## Supplementary Information

Below is the link to the electronic supplementary material.

Supplementary file1 (DOCX 72 KB)

## Data Availability

Due to the sensitive nature of the data, the data cannot be published on a public data repository. Requests to access the datasets should be directed to m.thoma@psychologie.uzh.ch.
