# Supplementary material for: Predicting Intraindividual Change in Satisfaction with Life During COVID-19: A Prospective Study of Swiss Older Adults with Differing Levels of Childhood Adversity
Source: J Happiness Stud. 2024 Jul 25;25(6):73. doi: 10.1007/s10902-024-00791-2 (PMC11272812; doi:10.1007/s10902-024-00791-2)
Supplement: Supplementary file 1 — Supplementary file1 (DOCX 72 KB) [file 10902_2024_791_MOESM1_ESM.docx]

**Supplementary Material Table 1:** Comparison of excluded and included participants.

*Note.* Values for meaning in life and adverse childhood experiences could not be computed as only one of the excluded participants answered these questionnaires.

|  | **Participants** | | | |
| --- | --- | --- | --- | --- |
|  | Excluded  (*n* = 12) | | Included  (*n* = 231) | |
| **Variables** | **%** | ***n*** | **%** | ***n*** |
| Gender (% of women) | 60% | 7 | 54% | 123 |
|  | ***M*** | ***SD*** | ***M*** | ***SD*** |
| Age | 77.2 | 16.2 | 70.4 | 10.7 |
| Emotion Regulation – Cognitive Reappraisal | 26.2 | 5.8 | 27.7 | 7.3 |
| Emotion Regulation – Expressive Suppression | 14.3 | 2.9 | 15.8 | 5.7 |
| Subjective Socio-Economic Status | 5.3 | 2.7 | 5.5 | 2.1 |

**Supplementary Material Table 2:** Intraindividual change in satisfaction with life predicted by adverse childhood experiences.

| **Risk Group (*n* = 111)** | | | | **Control Group (*n* = 120)** | | |
| --- | --- | --- | --- | --- | --- | --- |
| **Fixed Effects** | **Estimates** | ***SE*** | **95% CI** | **Estimates** | ***SE*** | **95% CI** |
| Intercept | -0.01 | 0.18 | -0.38; 0.35 | -0.01 | 0.14 | -0.29; 0.27 |
| Slope | -20.2** | 4.22 | -28.58; -20.27 | 2.8 | 4.24 | -5.44; 11.22 |
| Time × Age | 0.24** | 0.06 | 0.12; 0.24 | -0.08 | 0.05 | -0.16; 0.18 |
| Time × Gender | 0.90 | 1.32 | -1.69; 0.90 | 0.48 | 1.09 | -1.66; 2.64 |
| Time × ACE | 0.10 | 0.21 | -0.30; 0.10 | -0.30 | 0.19 | -0.68; 0.06 |
| **Random Effects** |  |  |  |  |  |  |
| Intercept |  | 0.45* | 0.30; 0.67 |  | 0.39 | 0.31; 0.51 |
| Slope |  | 0.09* | 0.06; 0.13 |  | 0.08 | 0.06; 0.10 |
| Residual |  | 0.68 |  |  | 0.54 | 0.51; 0.56 |
| -2LL |  | -529 |  |  | -766 |  |
| AIC |  | 1092 |  |  | 1564 |  |

*Note. SE =* standard errors; 95% CI *=* 95% confidence intervals; -2LL = 2 Log likelihood; AIC = Akaike’s Information Criterion; **p* < .05

**Supplementary Material Table 3:** Intraindividual change in satisfaction with life predicted by emotion regulation.

| **Risk Group (*n* = 111)** | | | | **Control Group (*n* = 120)** | | |
| --- | --- | --- | --- | --- | --- | --- |
| **Fixed Effects** | **Estimates** | ***SE*** | **95% CI** | **Estimates** | ***SE*** | **95% CI** |
| Intercept | -0.02 | 0.14 | -0.31; 0.26 | 0.001 | 0.14 | -0.28; 0.29 |
| Slope | -12.02* | 4.34 | -20.54; -3.50 | 4.96 | 4.46 | -3.80; 13.72 |
| Time × Age | 0.11 | 0.06 | -0.01; 0.23 | -0.11 | 0.06 | -0.23; 0.01 |
| Time × Gender | 1.36 | 1.36 | -3.35; 1.99 | 0.53 | 1.66 | -2.12; 2.17 |
| Time × CR | 0.15 | 0.09 | -0.03; 0.34 | 0.12 | 0.07 | -0.02; 0.27 |
| Time × ES | -0.16 | 0.12 | -0.41; 0.07 | 0.03 | 0.10 | -0.16; 0.24 |
| **Random Effects** |  |  |  |  |  |  |
| Intercept |  | 0.55* | 0.42; 0.70 |  | 0.43* | 0.34; 0.55 |
| Slope |  | 0.12* | 0.09; 0.15 |  | 0.09* | 0.07; 0.12 |
| Residual |  | 0.68* | 0.64; 0.72 |  | 0.53* | 0.51; 0.56 |
| -2LL |  | -950 | 0.64; 0.72 |  | -816 |  |
| AIC |  | 1941 |  |  | 1670 |  |

*Note. SE =* standard errors; 95% CI *=* 95% confidence intervals; -2LL = 2 Log likelihood; AIC = Akaike’s Information Criterion; **p* < .05
